# Narrative reconstruction therapy for prolonged grief disorder—rationale and case study

**DOI:** 10.3402/ejpt.v7.30687

**Published:** 2016-05-04

**Authors:** Tuvia Peri, Ilanit Hasson-Ohayon, Sharon Garber, Rivka Tuval-Mashiach, Paul A. Boelen

**Affiliations:** 1 Department of Psychology, Bar-Ilan University, Ramat Gan, Israel; 2 Department of Clinical and Health Psychology, Utrecht University, Utrecht, The Netherlands

**Keywords:** Narrative reconstruction, PGD, bereavement, PTSD, CBT, case study

## Abstract

**Background:**

Prolonged grief disorder (PGD) is a potentially disabling condition affecting approximately 10% of bereaved people. It has been suggested that the impaired integration of the loss memory, as expressed in recurrent memories of the loss and disorganization of memory, is involved in the development of PGD. Narrative reconstruction (NR), originally designed for the treatment of posttraumatic stress disorder (PTSD) in an integrative therapy module, and consisting of exposure to the loss memory, detailed written reconstruction of the loss memory narrative and an elaboration of the personal significance of that memory for the bereaved, has been shown to be effective in the treatment of intrusion symptoms.

**Objective:**

In light of findings that cognitive behavior therapy (CBT), including cognitive restructuring and exposure, is effective in the treatment of PGD, we suggest the implementation of a somewhat novel therapy module, NR, for the treatment of intrusive phenomena in bereaved patients.

**Method:**

The rationale for the implementation of NR for PGD and a case study of the treatment of a woman suffering from PGD after the death of her father are presented. Therapy took place in a university outpatient training clinic.

**Results:**

Evaluations conducted before and after treatment and at a 3-month follow-up demonstrated the effectiveness of NR in reducing symptoms of PGD and depression. The analysis of spontaneous narratives recorded before and after treatment showed an increased organization of the narratives.

**Conclusions:**

This case report demonstrates an adaptation of NR for the treatment of PGD. The results provide preliminary support for the effectiveness of NR for PGD. The significance of the study and its limitations are discussed.

**Highlights of the article:**

- Prolonged grief disorder (PGD) affects approximately ten percent of bereaved people.
- Narrative Reconstruction (NR), an integrative therapy module originally used for PTSD patients, was adapted for PGD.
- NR consists of exposure to the loss memory, detailed written reconstruction of the loss and an elaboration its significance and meaning for the bereaved.
- NR was demonstrated in a case study. It was well tolerated and effective in the treatment of PGD.

The loss of a loved one is a common stressor in the life of a human being (Parkes & Prigerson, 2013). Approximately 10% of bereaved people suffer from debilitating grief reactions, such as a painful yearning for the deceased, separation anxiety, difficulties accepting the loss, and difficulties engaging in new activities after the loss (Maercker & Lalor, 2012). When the symptoms last for at least 6 months and cause significant impairment in social and occupational functioning the syndrome is considered a mental disorder (Prigerson, Vanderwerker, & Maciejewski, 2008; Wittouck, Van Autreve, De Jaegere, Portzky, & Van Heeringen, 2011). Herein, we refer to this disorder as Prolonged Grief Disorder (PGD), a term previously proposed by key researchers in the field (Prigerson et al., 2009) and now proposed for the upcoming International Classification of Diseases, 11th Revision (Maercker et al., 2013). PGD differs from normal expected reactions to loss (Boelen & Van Den Bout, 2008), bereavement-related depression and anxiety (Boelen, Van Den Bout, & De Keijser, 2003), and major depression, adjustment disorder and posttraumatic stress disorder (PTSD) (for review, see Shear et al., 2011).

Although PTSD differs from PGD (Boelen, Van De Schoot, Van den Hout, De Keijser, & Van den Bout, 2010), significant similarities between these disorders are also evident. PGD is characterized by persistent yearning, preoccupation with the deceased and intrusive images (Prigerson et al., 2009), similar to the PTSD intrusion cluster. “Avoidance of reminders of the reality of the loss” and “numbness” are consistent with avoidance symptoms in PTSD. Bitterness and anger, together with difficulties moving forward in life, parallel the negative alterations in cognitions and moods in the new D cluster in the Diagnostic and Statistical Manual of Mental Disorders (DSM-5) criteria for PTSD (APA, 2013). The similarities between these two disorders have prompted the use of PTSD psychotherapy methods, primarily cognitive behavioral therapy (CBT) for PGD patients (Bryant et al., 2014; Shear et al., 2014; for review, see Rosner, 2015). These interventions have included cognitive behavioral techniques such as exposure and cognitive restructuring (Boelen, De Keijser, Van Den Hout, & Van Den Bout, 2007), integrative CBT (Rosner, Kotoucová, & Hagl, 2014), behavioral activation (Papa, Sewell, Garrison-Diehn, & Rummel, 2013), a combination of CBT with interpersonal elements (Shear, Frank, Houck, & Reynolds, 2005) and writing assignments based on Pennebaker's writing paradigm (Pennebaker, 1989; Wagner, Knaevelsrud, & Maercker, 2006). We may conclude that exposure to painful aspects of the loss is part of most CBT interventions for PGD, a conclusion that is supported by recent controlled studies (Bryant et al., 2014; Shear et al., 2014).

Based on a constructivist approach, Neimeyer presented a narrative-based treatment for PGD, involving meaning-reconstruction and an assimilation of the loss memory into one's personal narrative (Neimeyer, 2006, 2012; Neimeyer, Burke, Mackay, & van Dyke Stringer, 2010). This intervention incorporates journal writing (Lichtenthal & Neimeyer, 2012), retelling the story of the death (Neimeyer, 2012) and various other techniques based on the constructivist approach (Neimeyer et al., 2010). There is preliminary evidence that this technique is effective in reducing PGD symptoms (Gerrish, Steed, & Neimeyer, 2010; Gillies, Neimeyer, & Milman, 2014).

Based on the promising findings regarding exposure-based CBT and meaning-construction through narration, we suggest the implementation of Narrative Reconstruction (NR), an integrative treatment module originally developed for PTSD patients which incorporates exposure and narration, for the treatment of PGD. The adjustment of NR for PGD is described and demonstrated through a detailed evidence-based case report.

## The rationale for the implementation of NR to treat PGD

NR is a time-limited (12–16 sessions) integrative intervention which has been shown to be effective in reducing both PTSD and depression symptoms (Peri, 2004; Peri & Gofman, 2014; Peri, Gofman, & Vidan, 2013). NR involves (1) exposure to the traumatic event through a retelling of the event, (2) systematic reconstruction and reorganization of the traumatic memory into a coherent written narrative, (3) integration of the traumatic memory with other autobiographical memories and (4) psychodynamic references to the subjective personal meaning of the event for the patient, associated with his/her past experiences.

NR draws from empirical findings regarding incoherence and disorganization of trauma narratives (Foa, Molnar, & Cashman, 1995; Jelinek, Randjbar, Seifert, Kellner, & Moritz, 2009; O'Kearney, Hunt, & Wallace, 2011) and from conceptualizations of intrusion symptoms as resulting from the lack of integration of the traumatic experience into the autobiographical memory network (Brewin, 2011; Brewin, Gregory, Lipton, & Burgess, 2010). Recent studies of PGD have also focused on the impaired integration of the death narrative and memories of the deceased into the autobiographical memory (Barbosa, Sá, & Carlos Rocha, 2014; Boelen, Huntjens, Van Deursen, & Van Den Hout, 2012; Maccallum & Bryant, 2010; Neimeyer, Klass, & Dennis, 2014). The Self-Memory System Model (Brewin et al., 2010; Conway, Singer, & Tagini, 2004) further illustrates the role of impaired integration. According to this model, painful memories inconsistent with the individual's current goals remain in the working memory in the form of repeated intrusions rather than being integrated into the larger autobiographical networks (Boelen, Van Den Bout, & Van Den Hout, 2006; Maccallum & Bryant, 2010). Additionally, difficulties in building a coherent narrative and a personal meaning of the loss can lead to the recurrent yearning for the bereaved in PGD patients (Gillies et al., 2014).

Given the similarities in intrusion symptoms between PTSD and PGD and in the theoretical explanations accounting for these similarities, a revised version of NR for PGD was offered as an intervention for a patient who lost her father to cancer approximately 1 year before she began therapy.

### NR treatment outline

NR is based on the systematic reconstruction of the loss memory narrative along a chronological timeline. The patient is asked to describe what he/she was doing, seeing, hearing, and feeling at the time the event took place, minute by minute. The patient is also asked to relate his/her thoughts and emotions as the story unfolds, and at each session the therapist types everything the patient says. During the NR gaps are recorded, and an effort is made to return to those gaps during future sessions or when the patient recalls the missing information. The therapist might ask questions, clarify facts and ask for past memories associated with those facts. The therapist and the patient work together to uncover the meaning and significance of the loss event for the patient. Moments of personal significance are identified, dissociated facts and feelings are revealed, and the memory of the loss is actively connected with the patient's other, earlier memories.

At the last session, the patient is handed a printed copy of the final version of his/her narrative, and the therapist and the patient review the full narrative once more. The session concludes with a review of the changes in the patient's emotional state, attained through therapy.

### Modification of the NR protocol for PGD

The adaptation of the NR protocol for use with PGD patients required a consideration of the differences between PTSD and PGD regarding intrusion and avoidance symptoms. PTSD is characterized by intrusive memories of the traumatic event primarily accompanied by fear and anxiety, while in PGD the recurrent uncontrolled thoughts are frequently associated not only with the death itself but also with good things, such as positive memories of the deceased (Boelen et al., 2006; Boelen & Huntjens, 2008). In addition, PTSD patients avoid reminders of the trauma in an effort to control fear responses, while PGD patients yearn for the deceased (Shear et al., 2011) and avoid acknowledgement of the reality of their loss (Boelen et al., 2006; Shear et al., 2005). Hence, the choice of the event with which to associate the NR is not as clear as it would be after a more circumscribed traumatic event.

As the memory of the event of the death itself is not always central to the impaired memories and thoughts of PGD patients (Boelen & Huntjens, 2008), we suggested that the therapist identify the memories that were most distressing for the patient as expressed in his/her recurrent painful thoughts, including (1) memories associated with either positive or negative, yet meaningful, moments with the loved one before his/her death; (2) memories associated with the realization that the loved one is truly dead and gone (e.g., the moment the police informed the parent that his/her child was killed in an accident); and (3) memories that represent some meaningful and emotional moment after the loss (e.g., first meeting with the mother after the death of a brother).

In the current case, we selected a memory that repeatedly entered the patient's awareness in an uncontrolled manner. We assumed that the recurrent nature of this memory indicated the impaired integration of this memory into the patient's autobiographical memory network, reflecting his/her difficulty in accepting the reality of the loss (Boelen, 2010) or unresolved conflicts in his/her relationship with the deceased (Hagman, 2001).

## The current case study

In this work we present a case study demonstrating the implementation of modified NR for a PGD patient suffering from intrusive memories. We describe the therapy process and the changes following the intervention as measured before treatment, after treatment and at the 3-month follow-up. Spontaneous narratives of the memory of the event before and after treatment were recorded and analyzed to trace organizational changes (Foa et al., 1995; Halligan, Michael, Clark, & Ehlers, 2003). We hypothesized that the effects of NR would be reflected in improvement in distress measures and in enhanced organization of the trauma memory narrative following the intervention.

### The patient

Gail was a 35-year-old woman and the youngest of three children. At the time of her referral, she was graduating with a master's degree in social sciences and had been living with her boyfriend for 2 years. Gail's father died of cancer a year and a half before she sought treatment.

She complained of having trouble moving forward with her life due to recurrent painful memories of her father and intense longing for him together with anxieties regarding her ability to survive without him. She reported having a hard time accepting the loss and acknowledging the irreversibility of the death. There were moments of numbness and detachment from the memory of the absence of her father. She also complained that her mood swings caused her to fear being alone and made her dependent on her partner to a degree that affected their relationship. During the intake, she was interviewed and diagnosed as suffering from PGD according to the criteria of Prigerson et al. (2009).

## Measures

### The Structured Clinical Interview for DSM-IV

The Structured Clinical Interview for DSM-IV (SCID; First & Gibbon, 1997) was used to assess possible comorbid axis I disorders.

### Prolonged Grief Disorder Inventory

Prolonged Grief Disorder Inventory (PG-13; Prigerson et al., 2009) assesses the severity of prolonged grief symptoms over the past month. The items address separation distress and cognitive, emotional and behavioral symptoms. Each of these items is rated on a 5-point scale for frequency (1=*not at all* to 5=*several times per day*) and severity (1=*not at all* to 5=*overwhelmingly*). A total PGD severity score is derived from totaling the items. A prior study showed that the measure has good internal consistency (*α*=0.92) (Field et al., 2014).

### Clinician-Administered PTSD Scale

This scale is a comprehensive structured interview comprising 30 items, including 17 items that assess DSM-IV PTSD symptoms. The Clinician-Administered PTSD Scale (CAPS; Blake et al., 1995) shows excellent convergent and discriminant validity, diagnostic utility and sensitivity to clinical change (Weathers, Keane, & Davidson, 2001). The Hebrew translation has been used extensively in previous studies in Israel (Shalev, Freedman, Peri, Brandes, & Sahar, 1997).

### Beck Depression Inventory

The Beck Depression Inventory (BDI; Beck & Steer, 1993) is a 21-item self-report questionnaire tapping cognitive, behavioral and affective facets of depression with high reliability and validity. The BDI showed high validity and reliability scores and high internal consistency (*α*=0.81 to 0.86) (Beck, Steer, & Carbin, 1988). The Hebrew version of the BDI has been used extensively in studies in Israel and has shown good to excellent internal reliability (Levav, 2009, p. 66).

### Narrative organization analysis

A spontaneous narrative of the most distressing recurrent memory associated with the loss was obtained and recorded according to the procedure described in studies of PTSD patients (Jelinek et al., 2009) before and after therapy. The audiotaped narratives were then transcribed verbatim and coded by two raters blind to the diagnostic status. When the raters disagreed, the final decision was made by one of the authors of this work (SG). The narratives were coded according to scoring rules first introduced by Foa et al. (1995), modified by Halligan et al. (2003), and widely used in studies of PTSD (Jelinek et al., 2009). Accordingly, the beginning and end of the narrative were defined, and narratives were then divided into utterances assigned to four different categories: repetitions, disorganized thoughts, organized thoughts, and not coded. Finally, the percentage of each category was calculated to enable a comparison with former studies.

## Procedure

The case study presented herein was conducted at a university training clinic in Israel and approved by the university's institutional review board (IRB).

Gail applied for treatment after seeing an online advertisement. At her intake meeting, she received a description of the therapy and the accompanying evaluations, and she signed an informed consent form. Gail was also told that she could withdraw from the study at any point. A clinical psychology student who had been trained in the use of the different research tools conducted the assessment, which included the SCID, BDI, CAPS, PG-13 and the recording of a spontaneous narrative of the most distressing recurrent memory associated with the loss.

After the conclusion of the treatment and follow-up evaluations, Gail was asked for permission to publish a report of her therapy, and she agreed. Significant personal details were disguised to avoid her identification. A final draft was shown to Gail, and she further approved the publication of this study.

### Treatment course

Gail's therapist, SG, was an intern in clinical psychology and participated in weekly supervision sessions with TP, one of the developers of the NR protocol. Upon first evaluation, Gail met the criteria for diagnosis of PGD (PG-13=42) and scored above clinical cut-off points on the BDI (22) and the CAPS (50). Gail was not diagnosed with PTSD because she did not meet the A criterion of a traumatic event, yet the high CAPS score demonstrated intensive intrusions, avoidance and negative emotions after the loss of her father.

At the first meeting Gail provided what she perceived as the most intrusive event associated with the loss of her father. This event revolved around meeting with her father's oncologist approximately 6 months after his illness was diagnosed. At this meeting, her father was informed of metastases in his brain, and he reacted intensely, screaming and crying. This event is hereafter denoted as the Grief Event (GE).

#### Session 1

The first session was aimed at familiarizing Gail with the therapy and learning more about her background. She described her parents’ relationship as “harsh, loaded with criticism, mutual blaming and anger.” Gail remembered being closer to her father, who had difficulty being alone. She recalled her father spending time with her as a child, and as she grew older, he consulted with her on different topics. He used to share “family secrets” with her. In the period prior to the diagnosis of his illness, Gail distanced herself from her father, wanting more independence. She felt that this relationship was too intense and placed a burden on her, limiting her ability to develop other areas of her life.

Patient: On February 10, 2009, Dad told me they discovered a mass in his lungs, which they would need to examine. A few days before that, he had wanted to come and meet me. I tried to avoid the meeting … every time he suggested coming I gave a different excuse … at that time I was very busy with a new relationship that had just come into my life … my father felt I was keeping my distance from him. He asked me to come with him to the biopsy. It was weighing on me … in the end it happened on a day when I could not leave work and he went with my mother.

Gail revealed that this “separation” was not easy and was accompanied by guilt. When she was told about the discovery of his illness, she felt that she had abandoned him or possibly even caused his illness.

#### Sessions 2–6: “Any time I was angry or distant, I felt guilty”

The second meeting denoted the actual beginning of the work on the GE memory. The GE occurred approximately 6 months after the initial cancer diagnosis. Before that, Gail and her family had been rather optimistic about her father's recovery, as he had undergone one successful surgery and was awaiting another. The GE took place during the period between the discovery of the metastases in her father's brain and the meeting with his physician a few days later, on Sunday, when he himself was informed of the bad news.

The beginning of the reconstruction with Gail was extremely challenging. It was hard for her to focus and remember details of the event. Her speech was slow and detached; it seemed as if she was in a state of great anxiety and coped with it through dissociation. She often yawned, and the more distressing the emotional content the more she appeared bored and sleepy. The following vignette illustrates the therapist's effort to address Gail's emotional detachment:

Therapist: What do you remember of the event? Tell me how everything began. Please describe the background, the weather, your thoughts and feelings at that time. Follow a timeline and tell me as things happened minute by minute.

Patient: It was Friday … I don't remember very much ….

Therapist: Take your time …

Patient: I don't remember … I really don't remember … (yawning, looks detached)

Therapist: You may close your eyes. Try to remember. What did you wear? What did the room look like?

Patient: What did I wear? I don't remember … the room was small, at the end of the corridor … I don't remember … It was Friday … My boyfriend and I … we had intended to spend the weekend up north … so I had to cancel it … I think I was upset as once again I had to give up my life for my father … It's terrible what I am saying …

Therapist: It's natural. You wanted to invest in your new relationship. Spending a night in the hospital instead was frustrating and upsetting.

Patient: It was Friday. I remember that it was a chilly day … I believe I was wearing a sweater … it was brown … that morning my brother called. He said that they (the doctors) discovered metastases in Dad's brain … The doctor said that they would hold a meeting on Sunday to discuss his condition and his medical treatment … we, the children, decided not to tell Dad until Sunday. It was my shift … I remember my father … we were sitting in the room, talking, he was in a good mood … he was on steroids to ease his pain and they lifted his mood … I remember that it upset me that he behaved as if he were recovering … he was kind of optimistic … his attitude was not connected to reality … such a lack of awareness …

Therapist: What made you so angry?

Therapist: I don't know what upset me … maybe because I was aware of his condition … It was difficult for me to be with him in this unrealistic place, this lack of awareness … It wasn't real …

Therapist: That there was a gap between what he knew and what you knew? Because you were hiding the facts from him?

Patient: Yes, exactly … this decision to conceal from him … It was difficult …

Gail seemed to be flooded with intense feelings of guilt towards her father, exacerbated by the additional layer of guilt that stemmed from her feelings of rage towards him. This emotional turmoil caused her great anxiety and limited her ability to express other emotions. To address this difficulty, the therapist suggested that anger and aggression were legitimate parts of any close relationship with family members and encouraged Gail to express her feelings freely. This acknowledgment enabled Gail to go on and describe the meeting with the doctor:

Patient: Then the doctor arrived. She was very rigid and tough. She came in with this terrible attitude of concealing any emotion. She came in with the intention to get it over with quickly. I remember that she entered the room … I realized that my father's face had changed. I saw on his face a kind of fear, of terror. Suddenly his good spirits disappeared, he instantly changed … and then the doctor said emotionlessly: “There are metastases that spread to the brain … there are two options, either to go for focused radiation therapy or to go for chemotherapy.” That was it. She left the room and there was a kind of shock, of silence, especially his. I remember that he started to cry. He said that he wanted to die. I sat down in a chair, weeping. My aunt was in the chair next to him, trying to calm him, and she said to me: “Why are you crying? I am trying to calm him down and you are making it worse.” She even asked me to leave the room.

#### Sessions 7–11: “I remember being jealous … things were out there on the table. They knew they were approaching the end and talked about it. We were not like this”

During this phase, treatment was focused on processing Gail's relationship with her father as manifested in the narrative of the GE memory. The therapist applied psychodynamic techniques to identify and understand the personal significance of the GE for Gail. Gail described how unbearable it was for her to pretend to be happy and optimistic while concealing from her father the true nature of his condition. This unbearable situation was at the center of Gail's complicated grief experience. Concurrently, the therapist became aware of anger and guilt feelings within the therapeutic dyad. Although Gail verbally expressed satisfaction with the therapy, the therapist felt that beneath the surface she was disappointed and angry with her. The following dialogue during the eighth session demonstrates these complicated emotional states:

Therapist: You talk about the painful emotions that came from having to conceal the truth from your father, and about your wish that things “will be on the table.” I think that in this context it is very important that here, between us, things will be on the table so that you can share with me your feelings about the treatment, about me, about our relationship…

Patient: (looks embarrassed) Sometimes I think … how do we know that it will be good and not bad? … to talk about all this …

Therapist: I think that that is a very good question … It's important that things proceed at your own pace so that you can voice your doubts openly.

Gail further revealed that these feelings were associated with the fact that the therapy took place as part of a time-limited experimental research project, including frequent evaluations that seemed to have the study's best interests—rather than hers—at heart. The therapist, knowing that the therapy was indeed part of a research project, experienced some guilt. Despite having received both Gail's informed consent and the IRB approval, the therapist worried that the painful process of NR might turn out not to be helpful to Gail. In supervision, the therapist came to understand that these feelings reflected and reenacted the internalized dynamic of conflicting loyalties between Gail and her father.

At the next session, while reading through the written narrative, Gail elaborated on her relationship with her father and was overwhelmed by early memories and associations. She described a host of family secrets ranging from the small and insignificant to the large and very significant, including incidents of betrayal and even extra-marital relationships involving her father, of which only Gail was aware. Hiding information, which now played a major role in Gail's current difficulties, seemed to have been a central family theme throughout her life.

Patient: My uncles left and I stayed with Dad. I remember that he said to me: “What happened? Did something bad happen with you?” I … felt very bad. I knew about the metastases in his brain … It was difficult … (Silence…)

Therapist: Was this experience of having to conceal something from your father familiar to you from before?

Patient: Sure, our family's foundations were built on concealment … as a child my father used to share with me his difficulties with Mom … he even shared with me an affair he had … we were always kind of allies … many times it put me in a very uncomfortable position …

Therapist: You had to choose your loyalties …

Patient: Exactly …

Therapist: … And you had to cooperate with hiding any information that may have harmed your parents’ relationship, and now here again you had to conceal information from your father.

Patient: Yes …

Gail recalled that in a room near her father's (in the hospital) there was a woman close to death. The daughters of this woman entered her room one by one to say goodbye to her. Although Gail's description of this episode was heartbreaking, she talked about it enviously:

Patient: I remember feeling jealous that things were “on the table.” They knew that they were approaching the end and they talked about it. It wasn't like this in my family. We had secrets. Dad hid information from Mom, Mom hid things from Dad, and at that moment I also hid the truth from him.

At the end of this session, Gail's body language had softened, conveying a sense of inner peace and calm. She thanked her therapist for the opportunity to share her feelings openly. It was apparent that her successful experience in conveying her complicated emotional attitude towards the therapist strengthened their alliance and enabled Gail to face painful GE memories.

#### Sessions 12–14: “Suddenly there was a shift between anger and intimacy”

The last phase of the therapy involved the completion of the narrative. The narrative ended on a slightly more pleasant note than it began. When all the family members gathered around the father's bed, Gail reported feeling “somewhat less lonely” and having a sense of relief.

Therapist: Finally things were “on the table”…

Patient: Yes … It was genuine, especially in comparison to the false communication that was there before …

Therapist: And then you could be together to cope with things …

Patient: Exactly … I remember that I came in and hugged him … I really cried. We cried together. It did not last long but there was this feeling of relief … for both of us. After a few minutes, Mom arrived. As she entered the room I saw him looking at her as if to gauge her feelings. At that moment it occurred to me that at the end of the day she was important to him … it occurred to me that, after all, there was some love between them …

Gail reported that along with the progress in the therapy she also experienced some stabilization in some of her other relationships. Gail associated her improved relationship with her boyfriend, and her ability to think about and discuss the future of their relationship, with the therapy process.

Gail was also more forgiving of herself, perceiving herself as having done the best she could in the circumstances. Her need for autonomy and distance from her father, together with her anger about his clinginess, were now recognized as legitimate and normal responses, not as egoism. These realizations also enabled Gail to mourn the loss of her father and to make room for the sorrow she had felt for him.

The final two sessions were dedicated to the full recitation of the narrative. Gail felt that this exercise gave her a newfound perspective and distance that made the experience more tolerable, “as if it is part of a broader story.” When the printed and bound copy was handed to her, she responded by saying, “Something that had been stuck inside me has started to move.”

## Outcome evaluation

Symptoms of PGD, BDI and PTSD were assessed before and after treatment, as well as at the 3-month follow-up (Table 1). The Reliable Change Index (RCI) for PG-13, CAPS and BDI were computed according to Jacobson and Truax (1991) using previous data sets to obtain the standard deviation and α coefficient for each measure.

**Table 1:** PG-13, CAPS and BDI before therapy, after therapy and at follow-up

Time of measurement	Before therapy	After therapy (RCI)	Follow-up (RCI)
Outcome variable			
PG-13	42	31 (2.66)	23 (4.59)
CAPS—total	50	27 (3.61)	13 (5.81)
CAPS—Intrusion	17	7 (2.66)	5 (3.19)
CAPS—Avoidance	25	14 (2.27)	6 (3.92)
CAPS—Hyper arousal	10	6 (1.23 NS)	2 (2.47)
BDI	22	15 (2.09)	11 (3.28)

RCI—Reliable change index was calculated according to Jacobson and Truax (1991) using the formula of Bauer, Lambert, and Nielsen (2004). Values larger than 1.96 are considered significant.

Gail no longer met the criteria for PGD on the PG-13 at the end of therapy or at follow-up. The RCI was computed using the SD (5.98) and α coefficient (0.76) from a sample of PGD patients in a previous study (Schaal, Elbert, & Neuner, 2009) indicating a clinically significant change (Table 1). On the BDI, Gail went from moderate depression to mild depression at the end of therapy, and to minimal depression at follow-up (Beck, Steer, & Brown, 1996). The RCI was calculated using the SD (6.33) and α coefficient (0.86) from a large community sample (Seggar, Lambert, & Hansen, 2002) indicating a significant change.

On the CAPS, there was a reduction from moderate symptomatology (40–59) before treatment, to mild/subthreshold (20–39) at the end of treatment, to asymptomatic (0–19) at follow-up (Weathers, Ruscio, & Keane, 1999). The RCIs for the total score and for the subscales were computed using the SDs from a large sample of PTSD patients (Monson et al., 2008; CAPS total=18.38; Intrusion=7.1; Avoidance=9.16; Hyperarousal=6.13) and α coefficients for the total score (0.94) and for the three subscales (0.86) from Blake et al. (1995), yielding significant changes for the total CAPS score and for the intrusion and avoidance subscales after treatment and at follow-up (Table 1).

An analysis of the change in organization of the spontaneous narratives based on Foa's coding protocol (Foa et al., 1995) showed a significant change as manifested by, a longer narrative (from 166 utterances before treatment to 400 utterances after treatment) and a more organized narrative as indicated by the decrease in the repetitions and disorganized thoughts and the increase in the percentage of organized thoughts (Table 2). RCIs were computed using SDs (2.7; 3; 4.3, respectively) and error estimates (0.94) from Foa et al. (1995) indicating a significant change in all scales.

**Table 2:** Organizational analysis of spontaneous narratives before and after therapy

	% Before therapy	% After therapy	RCI
Repetitions	3.01	0.75	2.42
Disorganized thoughts	9.63	3.5	5.90
Organized thoughts	3.01	8	3.35

RCI—Reliable change index was calculated according to Jacobson and Truax (1991) using the formula of Bauer et al. (2004). Values larger than 1.96 are considered significant.

## Discussion

This case report demonstrates an adaptation of NR, originally developed for PTSD (Peri & Gofman, 2014), for the treatment of PGD. The results provide preliminary support for the effectiveness of NR for PGD patients, as they show a clinically significant decrease in psychopathology measures after treatment and an additional decrease at 3-month follow-up. The improvement in PGD symptoms was associated with an increase in measures of narrative organization similar to the findings in PTSD treatment with NR (Vidan, Tuval-Mashiach, & Peri, 2014). These concurrent processes of symptom reduction and narrative changes are consistent with studies showing associations between the level of organization and coherence of the narratives and psychopathology in general (Greenberg & Angus, 2004) and with PTSD symptoms (Foa et al., 1995; Jelinek et al., 2009; Tuval-Mashiach et al., 2004), depression (Nelson & Horowitz, 2001) and grief (Barbosa et al., 2014).

NR is part of a larger group of interventions in which the retelling of the loss event effectively reduces PGD symptoms (Boelen et al., 2007; Maccallum & Bryant, 2011; Simon, 2013; Wagner, Knaevelsrud, & Maercker, 2006). Yet NR includes some novel characteristics. The story is narrated in a structured manner along a timeline and is recorded by the therapist and gradually reconstructed in front of the patient. This strategy provides the sessions with structure and dictates an appropriately measured pace for the revelation of painful memories, thereby creating a secure atmosphere. Furthermore, the process of reconstruction includes associations with other life events, elaboration on the personal meaning of the event and to some extent, elaboration of the emotional exchange between patient and therapist. It is a protocol which allows for flexibility and could therefore be specifically tailored for each patient, consistent with process-focused therapeutic approaches for bereaved individuals (Emmanuelle, Ryckebosch-Dayez, & Delespaux, 2010).

### Potential underlying mechanisms of NR with PGD patients

The improvement in symptoms along the enhancement of narrative organization might indicate that improved processing of the loss memory fosters integration of the loss into the autobiographical memory, thereby reducing the unremitting presence of this event in the working memory (Brewin, 2011, 2014). Incoherent and fragmented trauma narratives (Foa et al., 1995; Jelinek et al., 2009) were perceived in Brewin's revised Dual Representation Theory for PTSD as being the result of dissociation between sensory bound representations of the trauma from more elaborated and contextualized representations integrated in the autobiographical memory (Brewin, 2011, 2014). During the narration of the loss event, the integration and contextualization of this memory into the autobiographical memory is enhanced: “By retrieving the distressing images and holding them in conscious attention, stronger C-reps (contextualized representations) are formed that provide a context for the memory in time and place. Further, associations between corresponding C-reps and S-reps (sensory-bound representations) are strengthened. Now, when reminders are encountered, the memories retrieved are contextualized and are experienced as belonging to the past rather than being a source of danger in the present” (Brewin, 2014, p. 88).

Consistent with the Dual Representation Theory, Boelen and colleagues (Boelen, 2010; Boelen et al., 2006) proposed a cognitive behavioral model of prolonged grief in which the nucleus of the disorder is associated with poor integration of the loss into autobiographical knowledge. Boelen (2010) further suggested that the subjective “sense of unrealness” of the loss is also associated with poor integration of the loss. Our findings of reduced intrusive memories and enhanced narrative organization might represent increased recognition of the reality of the loss.

Neimeyer and colleagues (Neimeyer et al., 2014; Neimeyer, Prigerson, & Davies, 2002) have also shown that the importance of deriving meaning through narration of the loss might be associated with the contextualization and integration of the death of a loved one into a larger network of one's memories. Rosner (2015), as well, describes “integration of the new and changed relationship to the bereaved” as being an essential ingredient of successful treatments. We propose that through the structured reconstruction of the narrative and the elaboration of the patient's personal meaning of the loss event, including the complicated relationship he/she might have had with the deceased, the contextualization of the memory is enhanced. This enhancement then improves the integration of the loss into the autobiographical memory and increases the realization of the finality of the loved one's death, leading to improvement in symptoms.

### Limitations

While the current case report presents preliminary support for the adoption of NR for PGD, further validation in studies using larger samples is needed. Additionally, the lack of session-by-session assessment limits the identification of the potential contribution of specific interventions to the reduction of symptoms. Follow-up for extended periods could determine whether the changes attained during therapy are stable for longer periods afterwards. An additional limitation of our study is that NR is composed of both a memory organization process and a meaning-making process, but the unique contribution of each could not be differentiated in the current study. Only larger scale dismantling studies will help to clarify this question. It should also be said that not using other efficient elements such as behavior activation, which could add to its therapeutic effect, somewhat limits NR. Yet even after considering the abovementioned limitations, this case report demonstrates that NR is potentially an effective intervention for the treatment of PGD.

## Supplementary Material

Narrative reconstruction therapy for prolonged grief disorder—rationale and case study

Narrative reconstruction therapy for prolonged grief disorder—rationale and case study

## References

[CIT0001] American Psychiatric Association (APA), DSM-5 Task Force (2013). Diagnostic and statistical manual of mental disorders: DSM-5*™*.

[CIT0002] Barbosa V, Sá M, Carlos Rocha J (2014). Randomised controlled trial of a cognitive narrative intervention for complicated grief in widowhood. Aging & Mental Health.

[CIT0003] Bauer S, Lambert M.J, Nielsen S.L (2004). Clinical significance methods: A comparison of statistical techniques. Journal of Personality Assessment.

[CIT0004] Beck A.T, Steer R.A (1993). Beck Depression Inventory manual.

[CIT0005] Beck A.T, Steer R.A, Brown G.K (1996). Manual for the BDI-II.

[CIT0006] Beck A.T, Steer R.A, Carbin M.G (1988). Psychometric properties of the Beck Depression Inventory: Twenty-five years of evaluation. Clinical Psychology Review.

[CIT0007] Blake D.D, Weathers F.W, Nagy L.M, Kaloupek D.G, Gusman F.D, Charney D.S, Keane T.M (1995). The development of a clinician-administered PTSD scale. Journal of Traumatic Stress.

[CIT0008] Boelen P.A (2010). A sense of “unrealness” about the death of a loved-one: An exploratory study of its role in emotional complications among bereaved individuals. Applied Cognitive Psychology.

[CIT0009] Boelen P.A, De Keijser J, Van Den Hout M.A, Van Den Bout J (2007). Treatment of complicated grief: A comparison between cognitive-behavioral therapy and supportive counseling. Journal of Consulting and Clinical Psychology.

[CIT0010] Boelen P.A, Huntjens R.J (2008). Intrusive images in grief: An exploratory study. Clinical Psychology & Psychotherapy.

[CIT0011] Boelen P.A, Huntjens R.J, Van Deursen D.S, Van Den Hout M.A (2010). Autobiographical memory specificity and symptoms of complicated grief, depression, and posttraumatic stress disorder following loss. Journal of Behavior Therapy and Experimental Psychiatry.

[CIT0012] Boelen P.A, Van De Schoot R, Van Den Hout M.A, De Keijser J, Van Den Bout J (2010). Prolonged grief disorder, depression, and posttraumatic stress disorder are distinguishable syndromes. Journal of Affective Disorders.

[CIT0013] Boelen P.A, Van Den Bout J (2008). Complicated grief and uncomplicated grief are distinguishable constructs. Psychiatry Research.

[CIT0014] Boelen P.A, Van Den Bout J, De Keijser J (2003). Traumatic grief as a disorder distinct from bereavement-related depression and anxiety: A replication study with bereaved mental health care patients. American Journal of Psychiatry.

[CIT0015] Boelen P.A, Van Den Bout J, Van Den Hout M.A (2006). Negative cognitions and avoidance in emotional problems after bereavement: A prospective study. Behaviour Research and Therapy.

[CIT0016] Brewin C.R (2011). The nature and significance of memory disturbance in posttraumatic stress disorder. Annual Review of Clinical Psychology.

[CIT0017] Brewin C.R (2014). Episodic memory, perceptual memory, and their interaction: Foundations for a theory of posttraumatic stress disorder. Psychological Bulletin.

[CIT0018] Brewin C.R, Gregory J.D, Lipton M, Burgess N (2010). Intrusive images in psychological disorders: Characteristics, neural mechanisms, and treatment implications. Psychological Review.

[CIT0019] Bryant R.A, Kenny L, Joscelyne A, Rawson N, Maccallum F, Cahill C, Nickerson A, … (2014). Treating prolonged grief disorder: A randomized clinical trial. JAMA Psychiatry.

[CIT0020] Conway M.A, Singer J.A, Tagini A (2004). The self and autobiographical memory: Correspondence and coherence. Social Cognition.

[CIT0021] Emmanuelle Z.E.C.H, Ryckebosch-Dayez A, Delespaux E (2010). Improving the efficacy of intervention for bereaved individuals: Toward a process-focused psychotherapeutic perspective. Psychologica Belgica.

[CIT0022] Field N.P, Strasser J, Taing S, Horiuchi S, Chhim S, Packman W (2014). Prolonged grief following the recent death of a daughter among mothers who experienced distal losses during the Khmer Rouge era: Validity of the prolonged grief construct in Cambodia. Psychiatry Research.

[CIT0023] First M.B, Gibbon M (1997). User's guide for the structured clinical interview for DSM-IV axis I disorders SCID-I: Clinician version.

[CIT0024] Foa E.B, Molnar C, Cashman L (1995). Change in rape narratives during exposure therapy for posttraumatic stress disorder. Journal of Traumatic Stress.

[CIT0025] Gerrish N.J, Steed L.G, Neimeyer R.A (2010). Meaning reconstruction in bereaved mothers: A pilot study using the biographical grid method. Journal of Constructivist Psychology.

[CIT0026] Gillies J, Neimeyer R.A, Milman E (2014). The meaning of loss codebook: Construction of a system for analyzing meanings made in bereavement. Death Studies.

[CIT0027] Greenberg L.S, Angus L.E, Angus L.E, McLeod J (2004). The contributions of emotion processes to narrative change in psychotherapy: A dialectical constructivist approach. The handbook of narrative and psychotherapy: Practice, theory, and research.

[CIT0028] Hagman G, Neimeyer R (2001). Beyond decathexis: Toward a new psychoanalytic understanding and treatment of mourning. Meaning reconstruction and the experience of loss.

[CIT0029] Halligan S.L, Michael T, Clark D.M, Ehlers A (2003). Posttraumatic stress disorder following assault: The role of cognitive processing, trauma memory, and appraisals. Journal of Consulting and Clinical Psychology.

[CIT0030] Jacobson N.S, Truax P (1991). Clinical significance: A statistical approach to defining meaningful change in psychotherapy research. Journal of Consulting and Clinical Psychology.

[CIT0031] Jelinek L, Randjbar S, Seifert D, Kellner M, Moritz S (2009). The organization of autobiographical and nonautobiographical memory in posttraumatic stress disorder (PTSD). Journal of Abnormal Psychology.

[CIT0032] Levav I (2009). Psychiatric and behavioral disorders in Israel from epidemiology to mental health action.

[CIT0033] Lichtenthal W, Neimeyer R, Neimeyer R (2012). Directed journaling to facilitate meaning-making. Techniques of grief therapy: Creative practices for counselling the bereaved. Series in death, dying, and bereavement.

[CIT0034] Maccallum F, Bryant R.A (2010). Impaired autobiographical memory in complicated grief. Behaviour Research and Therapy.

[CIT0035] Maccallum F, Bryant R.A (2011). Autobiographical memory following cognitive behaviour therapy for complicated grief. Journal of Behavior Therapy and Experimental Psychiatry.

[CIT0036] Maercker A, Brewin C.R, Bryant R.A, Cloitre M, Reed G.M, Van Ommeren M, Saxena S, … (2013). Proposals for mental disorders specifically associated with stress in the International classification of diseases-11. The Lancet.

[CIT0037] Maercker A, Lalor J (2012). Diagnostic and clinical considerations in prolonged grief disorder. Dialogues in Clinical Neuroscience.

[CIT0038] Monson C.M, Gradus J.L, Young-Xu Y, Schnurr P.P, Price J.L, Schumm J.A (2008). Change in posttraumatic stress disorder symptoms: Do clinicians and patients agree?. Psychological Assessment.

[CIT0039] Neimeyer R.A (2006). Bereavement and the quest for meaning: Rewriting stories of loss and grief. Hellenic Journal of Psychology.

[CIT0040] Neimeyer R.A, Neimeyer R.A (2012). Retelling the narrative of the death. Techniques of grief therapy: Creative practices for counselling the bereavement. Series in death, dying, and bereavement.

[CIT0041] Neimeyer R.A, Burke L.A, Mackay M.M, Van Dyke Stringer J.G (2010). Grief therapy and the reconstruction of meaning: From principles to practice. Journal of Contemporary Psychotherapy.

[CIT0042] Neimeyer R.A, Klass D, Dennis M.R (2014). A social constructionist account of grief: Loss and the narration of meaning. Death Studies.

[CIT0043] Neimeyer R.A, Prigerson H.G, Davies B (2002). Mourning and meaning. American Behavioral Scientist.

[CIT0044] Nelson K.L, Horowitz L.M (2001). Narrative structure in recounted sad memories. Discourse Processes.

[CIT0045] O'Kearney R, Hunt A, Wallace N (2011). Integration and organization of trauma memories and posttraumatic symptoms. Journal of Traumatic Stress.

[CIT0046] Rosner R (2015). Prolonged grief: Setting the research agenda. European Journal of Psychotraumatology.

[CIT0047] Rosner R, Pfoh G, Kotoucová M, Hagl M (2014). Efficacy of an outpatient treatment for prolonged grief disorder: A randomized controlled clinical trial. Journal of Affective Disorders.

[CIT0048] Papa A, Sewell M.T, Garrison-Diehn C, Rummel C (2013). A randomized open trial assessing the feasibility of behavioral activation for pathological grief responding. Behavior Therapy.

[CIT0049] Parkes C.M, Prigerson H.G (2013). Bereavement: Studies of grief in adult life.

[CIT0050] Pennebaker J.W (1989). Confession, inhibition, and disease. Advances in Experimental Social Psychology.

[CIT0051] Peri T (2004). It was like in the cartoons”: From memory to traumatic memory and back. Psychotherapy and Psychoanalysis.

[CIT0052] Peri T, Gofman M (2014). Narrative reconstruction: An integrative intervention module for intrusive symptoms in PTSD patients. Psychological Trauma: Theory, Research, Practice, and Policy.

[CIT0053] Peri T, Gofman M, Vidan Z (2013). Changes in trauma narratives following narrative reconstruction therapy and their correlation with PTSD intrusion symptoms.

[CIT0054] Prigerson H.G, Horowitz M.J, Jacobs S.C, Parkes C.M, Aslan M, Goodkin K, Maciejewski P.K, … (2009). Prolonged grief disorder: Psychometric validation of criteria proposed for *DSM-V* and *ICD-11*. PLoS Medicine.

[CIT0055] Prigerson H.G, Vanderwerker L.C, Maciejewski P.K, Stroebe M.S, Hansson R.O, Schut H, Stroebe W (2008). A case for inclusion of prolonged grief disorder in DSM-V. Handbook of bereavement research and practice: Advances in theory and intervention.

[CIT0056] Schaal S, Elbert T, Neuner F (2009). Prolonged grief disorder and depression in widows due to the Rwandan Genocide. Omega: Journal of Death and Dying.

[CIT0057] Seggar L.B, Lambert M.J, Hansen N.B (2002). Assessing clinical significance: Application to the Beck Depression Inventory. Behavior Therapy.

[CIT0058] Shalev A.Y, Freedman S, Peri T, Brandes D, Sahar T (1997). Predicting PTSD in trauma survivors: Prospective evaluation of self-report and clinician-administered instruments. British Journal of Psychiatry: The Journal of Mental Science.

[CIT0059] Shear K, Frank E, Houck P.R, Reynolds C.F (2005). Treatment of complicated grief: A randomized controlled trial. JAMA: The Journal of the American Medical Association.

[CIT0060] Shear M.K, Simon N, Wall M, Zisook S, Neimeyer R, Duan N, First M, … (2011). Complicated grief and related bereavement issues for DSM-5. Depression and Anxiety.

[CIT0061] Shear M.K, Wang Y, Skritskaya N, Duan N, Mauro C, Ghesquiere A (2014). Treatment of complicated grief in elderly persons: A randomized clinical trial. JAMA Psychiatry.

[CIT0062] Simon N.M (2013). Treating complicated grief. JAMA: The Journal of the American Medical Association.

[CIT0063] Tuval-Mashiach R, Freedman S, Bargai N, Boker R, Hadar H, Shalev A.Y (2004). Coping with trauma: Narrative and cognitive perspectives. Psychiatry.

[CIT0064] Vidan Z, Tuval-Mashiach R, Peri T (2014). Changes in trauma narratives following narrative reconstruction psychotherapy and their correlation with PTSD symptoms.

[CIT0065] Wagner B, Knaevelsrud C, Maercker A (2006). Internet-based cognitive-behavioral therapy for complicated grief: A randomized controlled trial. Death Studies.

[CIT0066] Weathers F.W, Keane T.M, Davidson J.R (2001). Clinician-Administered PTSD Scale: A review of the first ten years of research. Depression and Anxiety.

[CIT0067] Weathers F.W, Ruscio A.M, Keane T.M (1999). Psychometric properties of nine scoring rules for the Clinician-Administered Posttraumatic Stress Disorder Scale. Psychological Assessment.

[CIT0068] Wittouck C, Van Autreve S, De Jaegere E, Portzky G, Van Heeringen K (2011). The prevention and treatment of complicated grief: A meta-analysis. Clinical Psychology Review.

