# Supplementary material for: Narrative reconstruction therapy for prolonged grief disorder—rationale and case study
Source: Eur J Psychotraumatol. 2016 May 4;7:10.3402/ejpt.v7.30687. doi: 10.3402/ejpt.v7.30687 (PMC4858499; doi:10.3402/ejpt.v7.30687)
Supplement: Narrative reconstruction therapy for prolonged grief disorder—rationale and case study [file EJPT-7-30687-s001.pdf]

Terapia oparta na rekonstrukcji narracyjnej dla pacjentów cierpiących na zespół przedłużonej żałoby (Prolonged Grief Disorder, PGD) - studium przypadku.

Tuvia Peri, Ilanit Hasson Ohayon, Sharon Garber, Rivka Tuval-Mashiach, Paul A. Boelen

Wprowadzenie: Zespół przedłużonej żałoby dotyka ok. 10% ludzi po stracie ukochanej osoby. Zauważono, że jednym z czynników utrzymujących to zaburzenie jest niemożność zintegrowania faktu straty ukochanej pamięci do struktur pamięci.Terapia oparta na rekonstrukcji narracyjnej, pierwotnie skonstruowana do leczenia PTSD, może być pomocnym środkiem w integracji pamięci po traumie straty, przeciwdziałającym przede wszytskim nasilonym symptomom intruzji.
Cel: Bazując na danych pochodzących z badań nad terapią poznawczo-behawioralną, proponujemy w tym artykule nowe podejście do leczenia zespołu przedłużonej żałoby- opartego na rekonstrukcji narracyjnej.
Metoda: Prezentujemy studium przypadku z wykorzystaniem tego rodzaju terapii w leczeniu kobiety po śmierci jej ojca.
Wyniki: Ocena stanu pacjentki dokonana 3 miesiące po zastosowaniu tego rodzaju terapii wykazała znaczący spadek nasilenia zespołu patologicznej żałoby, oraz obnizenia objawów depresji.
Konkluzje: Niniejszy artykuł wykazał skuteczność erapii oparta na rekonstrukcji narracyjnej dla pacjentów cierpiących na zespół przedłużonej żałoby

Słowa kluczowe: rekontrukcja narracyjna; PGD; żałoba; PTSD; CBT; studium przypadku.

Name of translator: Marcin Rzeszutek, University of Finance and Management in Warsaw, Poland

Citation: European Journal of Psychotraumatology 2016, **7**: 30687 - http://dx.doi.org/10.3402/ejpt.v7.30687
