# Supplementary material for: Narrative reconstruction therapy for prolonged grief disorder—rationale and case study
Source: Eur J Psychotraumatol. 2016 May 4;7:10.3402/ejpt.v7.30687. doi: 10.3402/ejpt.v7.30687 (PMC4858499; doi:10.3402/ejpt.v7.30687)
Supplement: Narrative reconstruction therapy for prolonged grief disorder—rationale and case study [file EJPT-7-30687-s002.pdf]

Название: Нарративная реконструктивная (НР) терапия для расстройства пролонгированного горя - обоснование и анализ случая из практики

Tuvia Peri, Ilanit Hasson Ohayon, Sharon Garber, Rivka Tuval-Mashiach, Paul A. Boelen
Тувиа Пери, Иланит Хассон Охаион, Шарон Гарбер, Ривка Тувал-Иашиач, Пол А. Боелен

Абстракт
Исходная информация: Расстройство затяжного (пролонгированного) горя (РЗГ) являеться состоянием, которое может вызвать нетрудоспособность человека и расспространено приблизительно среди 10% горюющих. Согласно существующим объяснениям, в развитии данного состояния играет роль неспособность интегрировать потерю в память/воспоминания, что проявляется в повторяющихся воспоминаниях о потере и дезинтеграции памяти. Метод Нарративной реконструкции (НР) созданный в свое время для лечения посттравматических страссовых расстройств в модуле интегративной терапии, состоящий в экспозиции воспоминаниям потрери, детальной письменной реконструкции нарратива памяти/воспоминаний потери и осознании личного значения этих воспоминаний для горюющего, показал эффективность в лечении симптомов вторжения (интрузии).
Цель: В свете того, что эффективность Когнитивно-поведенческой терапии (КПТ), включая когнитивное реконструирование и экпозицию, доказана в лечении РЗГ, мы предлагаем осуществление инновационного модуля терапии – НР, для лечения интрузивных феноменов среди горюющих пациентов.
Метод: В статье предлагается обоснование для применения НР случаях РЗГ, а также анализ случая лечения женщины страдающей от РЗГ в последствии смерти своего отца. Терапия была осуществлена в университетской тренинговой амбулаторной клинике.
Результаы: Оценка состояния пациента до и после лечения, также как и последующая оценка (проведенная по истечении трех месяцев с конца терапии) продемонстрировали эффективность НР в редукции симптомов РЗГ и депрессии. Анализ спотнтанных нарративов записанных до и после лечения выявил увеличивающуюся организованность нарративов.
Выводы: Анализ проделанной работы демонстрирует адаптацию НР к лечению РЗГ. Результаты предварительно поддерживают эффективность НР в лечении РЗГ. В статье также обсуждаются важность исследования и его ограничения.

Ключевые слова: Нарративная реконструкция, ПЗГ, горе/потеря, ПТСР, КПТ, анализ случая из практики

Name of translator: Jana (Darejan) Javakhishvili

Citation: European Journal of Psychotraumatology 2016, **7**: 30687 - http://dx.doi.org/10.3402/ejpt.v7.30687
,
